# Psychophysiological Reactivity, Postures and Movements among Academic Staff: A Comparison between Teleworking Days and Office Days

**DOI:** 10.3390/ijerph18189537

**Published:** 2021-09-10

**Authors:** Linda Widar, Birgitta Wiitavaara, Eva Boman, Marina Heiden

**Affiliations:** Centre for Musculoskeletal Research, Department of Occupational Health Sciences and Psychology, Faculty of Health and Occupational Studies, University of Gävle, SE-80176 Gävle, Sweden; birgitta.wiitavaara@hig.se (B.W.); eva.boman@hig.se (E.B.); marina.heiden@hig.se (M.H.)

**Keywords:** working from home, academics, workplace stress, recovery, physical activity, heart rate variability, occupational health

## Abstract

The aim of this study was to determine if psychophysiological activity, postures and movements differ during telework (i.e., work performed at home) and work performed at the conventional office. We performed twenty-four-hour pulse recordings and accelerometry measurements on 23 academic teaching and research staff during five consecutive workdays, with at least one day of telework. Additionally, we conducted salivary sampling during one day of telework, and one day of office work. Heart rate and heart rate variability indices, postural exposure and cortisol concentration were analyzed using repeated measures analysis of variance with Workplace and Time (i.e., before, during and after workhours) as within-subject effects. We found a significant interaction effect of Workplace and Time in heart rate variability indices and in the number of transitions between seated and standing postures. This shows more parasympathetic activity among academic teleworkers during telework than office work, which may indicate more relaxation during telework. They had an overall sedentary behavior at both workplaces but switched between sitting and standing more often during telework, which may be beneficial for their health.

## 1. Introduction

To practice work from other physical places than in the conventional office has changed working life [1,2,3,4,5,6]. On the one hand, telework is found to increase individual autonomy, work/life balance, work control and productivity and reduce work related stress [1,2,5,7,8,9]. On the other hand, telework is associated with increased stress, low boundary control (i.e., difficulty separating work and life domains), overtime work, lack of work motivation, and insufficient time for recovery [5,8,10,11,12,13,14,15]. Thus, research shows disparate results with respect to the effect of telework on staff health and well-being.

According to a report from Eurofound and the International Labour Office [9], telework is usually practiced by those with high education and/or organizational position in so-called “knowledge- and information organizations” such as IT-enterprises and universities [1,5,9,16,17]. The high adoption of telework among academic staff has been related to expanding work demands caused by digitalization, such as increased demands on staff availability, teaching and research performance [4,11,14,18,19,20,21]. In order to cope with this demanding work situation, academic staff may exceed normal working hours by compensating with their free time [4,11,19,20,21]. Studies on the academic work environment show that staff report high levels of stress and lack of time for relaxation, sleep disturbance, work/life interference and decreased levels of physical activity [4,11,18,19,20,22]. Regarding telework, Currie and Eveline [11] found that academics experienced difficulties separating work and private life and reported longer working hours and stress-related disorders. Moreover, a study on Swedish academics showed that staff who teleworked frequently (>3 days per week) perceived high stress related to indistinct organization and conflicts [15]. In contrast, Tustin [23] found that home-based academic teleworkers perceived lower levels of fatigue, more satisfaction with their work and experienced higher productivity than non-teleworkers.

To the authors’ knowledge, no study has investigated stress during telework among academics, using objective measurements. However, Lundberg and Lindfors [24] showed that white collar workers had lower cardiovascular reactivity during telework than during work at the conventional office. They also found that men had an elevated blood pressure in the evening after teleworking compared to women. They attributed their findings mostly to more diverged work tasks performed at the conventional office compared to more focused work at home [24]. Moreover, they suggested that differences in physical activity during telework and work at the conventional office could affect the results [24].

In a recent study, Arántzazu et al. [25] found that self-reported sedentary behavior was associated with psychosocial stress among female professors during telework. However, since self-reports of sedentary behavior may be biased [26,27], the effects of telework in physical activity and sedentary behavior should also be investigated using objective measurements. It has been shown that physical activity can reduce psychophysiological reactivity while sedentary behavior can increase it [28,29,30,31]. Because of this, measures of physical activity are often combined with measures of physiological reactivity (e.g., pulse recordings), to study stress in different work settings [26,29,32,33,34]. Several reports and studies emphasize the importance of research on postures and movements to understand health outcomes among teleworkers [1,5,9].

Studies on the occupational health aspects of telework is of high relevance in these days of rapid technological development and COVID-19 lockdowns. Academics are amongst the groups that practice telework to a high extent, but little is known about staffs’ physical behaviors and psychophysiological responses to such flexible work arrangements. We believe it is important to investigate these factors in order to get a deeper understanding of what risks and/or possibilities telework could entail for academic staffs’ health. In the present study, we therefore, aimed to determine if psychophysiological reactivity, postures and movements differ during telework (i.e., work performed at home) and work performed at the conventional office amongst academic teaching and research staff.

## 2. Methods

### 2.1. Study Sample

In a previous questionnaire study [15], respondents were asked about interest in participating in the present study. In total, 111 employees from six universities in different parts of Sweden reported their interest, and were contacted via e-mail. Eligible participants were employed as junior lecturers, senior lecturers or professors and engaged in teaching and/or research ≥50% of their working time. All participants had to regularly practice telework and do so at least one day during the measurement period. Participants with medical heart conditions and those who had retired were excluded leaving 108 subjects eligible for inclusion. Among those, 23 academic staff agreed to participate. Information about the study aim and procedures were given in writing and verbally during the recruitment and at the time of data collection. Participants gave their informed consent before data collection. The Regional Ethical Review Board in Uppsala, Sweden has approved the study (Reg. No. 2016/494).

### 2.2. Study Design and Procedures

The study comprised five consecutive workdays of accelerometry measurements, pulse recordings and daily visual analogue scale (VAS) ratings. During at least one of the five workdays, the participants teleworked and during at least one of the days they worked at the conventional office. Salivary samples were collected during one day of telework and one day of work at the conventional office. On each workday, the participants documented place and time for work and leisure activities, as well as work tasks performed, in a diary. The measurement period started on a Monday morning with individual 30-min sessions where one member from the research team (L.W.) informed about the study procedure by handing out and demonstrating the data collection materials, and attaching the accelerometry and pulse recording devices. At Friday night, the participants removed the recording devices. Data collection lasted from August 2018 to June 2019.

#### 2.2.1. Ratings of Stress, Fatigue and Recuperation

Stress, fatigue and recuperation were assessed on a 100 mm VAS scale. It ranged from 0 (= not at all) to 10 (= completely) and higher values indicated more stress, fatigue and recuperation. Participants rated their levels of stress and fatigue before and after each workday, and how recuperated they felt in the morning after each workday.

#### 2.2.2. Salivary Sampling

A self-administrated Salivette active sampling technique [35] was used to measure cortisol concentration in saliva (ng cortisol/mL saliva). It consists of a standard centrifugation tube containing a small cotton-swab that was actively chewed on for 30 s, or until it contained a sufficient amount of saliva. Thereafter, the cotton-swab is replaced into the sampling tube and stored in a freezer at −18 °C. Salivary samples were collected approximately every 3rd hour, 6 times a day, starting at 7 AM and continuing throughout the day until 10 PM. Participants were instructed to refrain from nicotine, coffee and alcoholic beverages, and vigorous physical activity one hour before providing their salivary samples [35]. Each sampling tube was marked with a unique number.

#### 2.2.3. Pulse Recordings

Firstbeat Bodyguard 2 pulse recording device (Firstbeat Technologies Ltd., Jyväskylä, Finland) was used to determine psychophysiological reactivity by sampling heart rate (beats per minute (bpm)) and heart rate variability (beat-to-beat intervals (RRI)) continuously at 1000 Hz [36,37]. The pulse recording device were attached with electrodes to the participants’ upper right chest area under the collarbone, and to the lower left chest area on the rib cage. Participants were instructed to detach the pulse recording device before coming into contact with water (i.e., shower/bath) and reattach it when dry. Date and time for detach- and reattaching were noted in participants’ diaries.

#### 2.2.4. Accelerometry Measurements

Physical activity and arm elevation were assessed using two AX3 accelerometers (Axivity Ltd., Newcastle, UK) [38]. They were attached to the skin surface with adhesive tape distal on the deltoid muscle bracket and on mid quadriceps on the dominant side of the body. The accelerometers were then secured by plastic adhesive film. Data were sampled continuously at 25 Hz. In order to synchronize the equipment, participants were asked to perform reference measurements at the start and at the end of the measurement period, as well as once a day. This was done by standing in an upright position with arms alongside the body for 10 s, followed by a jump while remaining in the same position and another 10 s of standing still [38,39].

### 2.3. Data Processing

Salivary samples were thawed, vortexed and centrifuged at 1500× *g* for 15 min in order to remove particle matters that may contaminate and affect the estimated cortisol concentration values. Concentration of salivary cortisol was then analyzed with a Salimetrics Enzyme immunoassay kit according to customary procedures [35].

The AX3 OMGUI software (Axivity Ltd., Newcastle, UK) was used for downloading pulse recording data from Firstbeat Bodyguard 2. Data were then exported to the Firstbeat SPORTS 4.7 software (Firstbeat Technologies Ltd., Jyväskylä, Finland) and scanned through an artifact detection filter to remove falsely detected, missed and premature heartbeats [36]. Pulse recording data were then imported to Spike version 9 for Windows (Cambridge Electronic Ltd., Cambridge, UK) and screened for errors using custom algorithms in Matlab software. The following variables were calculated based on non-overlapping one-minute windows of pulse recordings: heart rate (bpm); heart rate variability in time domains (ms): the standard deviation of RRI (SDNN); the root mean squared successive differences of RRI (rMSSD); heart rate variability in frequency domains (ms^2^): low frequency (LF) (0.04–0.15 Hz); high frequency (HF) (0.15–0.40 Hz). Among these variables, rMSSD and HF are indexes of vagal control and reflect parasympathetic reactivity; LF reflects baroreceptor activity influenced by sympathetic reactivity; SDNN reflects the cyclic components responsible for the variability in the period of recordings, i.e., overall heart rate variability. All variables were selected based on recommendations made in previous research [33,36,40].

Accelerometer data were downloaded (sample rate 25 Hz) with Acti4(a) software [41] and then exported to Spike version 8 for Windows where it was screened for errors. Subsequently, median arm angle and number of transitions between sitting/lying and standing were calculated. In addition, the average time spent in sedentary behaviors (i.e., sitting, lying) and other behaviors (e.g., standing, walking, running) were computed [26,27,41]. Since time spent in different behaviors are interdependent, compositional data analysis was used [42,43]. It involved calculating an isometric log-ratio (ilr) coordinate of the relative information between sedentary behaviors and the other behaviors (i.e., standing/walking/moving) that could be handled using standard statistical methods such as analysis of variance [26,33].

Diary entries were used to distinguish data for each Workplace and Time domain. For each participant, the variables were averaged across workdays spent at the conventional office and workdays teleworking from home, respectively. Workplace had two levels: Office and Telework. Office was defined as work performed during regular workhours at the conventional workplace. Telework was defined as work performed during regular workhours from the participants’ home. For accelerometry measures and pulse recordings, the Time variable was divided into leisure time before workhours (i.e., the time from awakening to the workday begins)*,* work during regular workhours (i.e., 8:00 AM to 17:00 PM excluding commuting time) and leisure time after workhours (i.e., the time from the end of the workday to going to sleep excluding overtime work). For salivary samplings, the Time variable were divided into six hours (7:00, 9:00, 12:00 AM; 15:00, 18:00, 22:00 PM).

### 2.4. Statistical Analysis

Statistical analyses were performed in IBM SPSS Statistics 22.0 (IBM, Armonk, NY, USA) for Windows. Sample characteristics are presented as proportions, means and standard deviations in Table 1. Differences in estimated stress and fatigue before and after workhours, and estimated recuperation in the morning after workhours, were compared between Office and Telework in paired sample t-tests. For remaining outcomes, repeated measures analyses of variance (ANOVA) were performed with Workplace (two levels) and Time (three and six levels) as within-subjects effect. The ANOVAs were performed with and without adjustment for commuting time [10,44,45], children living at home [14,44,46] and gender [10,22,47,48]. When the assumption of sphericity was not met, the Huynh-Feldt correction was used [49,50]. Level of significance was set to *p* < 0.05 in all tests.

## 3. Results

The final sample consisted of 23 participants. There was an equal distribution of men and women, age ranged between 27 and 61 years, and the calculated mean for body mass index (BMI) was 25.4 (Table 1). Among included professions, 56% were junior lecturers, 35% senior lecturers and 9% professors. According to information provided by the universities’ HR departments, the sample is representative for the population. Their main work tasks were teaching activities such as planning and giving lectures and communicating with students; and administrative and research activities such as reading, responding to emails and participating in meetings. Participants were experienced teleworkers as most of them teleworked several times/month or several times/week. Their average commuting time to work was 38.7 min (data available for *n* = 22) and the majority of the sample had children living at home. During the measurement period, participants performed telework on average 1.8 days and worked from their conventional office on average 3.2 days.

### 3.1. Staff Ratings of Stress, Fatigue and Recuperation

For VAS ratings (shown in mm) of stress, there were marginal differences in ratings before workhours (telework: M = 20; office: M = 27), relative to after workhours (telework: M = 19; office: M = 28) during teleworking days and office days. The differences in ratings of fatigue before (M = 27) relative to after (M = 33) telework, were slightly larger, and similar in size compared to office work (before: M = 35; after: M = 42). Ratings of recuperation in the morning after workhours showed that participants felt fairly recuperated before going to work regardless of where they would work (telework: M = 60; office: M = 52).

Paired sample t-tests showed no significant difference in the effect of Workplace for self-rated stress (*p* = 0.689) and fatigue (*p* = 0.842) before compared to after workhours or for recuperation in the morning after workhours (*p* = 0.151).

### 3.2. Psychophysiological Reactivity

**Figure 1:**
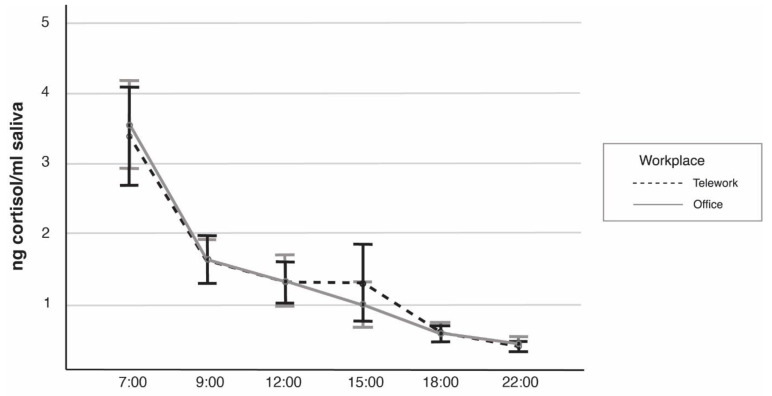
Estimated marginal means of Workplace (telework; office) and Time (hours of the day) from the repeated measures analysis of variance of ng cortisol/mL saliva (*n =* 23). Error bars represent 95% confidence interval.

For the salivary sampling, the repeated measures analysis of variance showed a significant effect of Time on cortisol concentration (*p* < 0.001, η_2_ = 1.000) but no interaction effect of Workplace and Time. Participants’ salivary cortisol showed a normal variation during the day at both workplaces with the highest concentration in the morning and a gradual decrease during the day (Figure 1).

**Figure 2:**
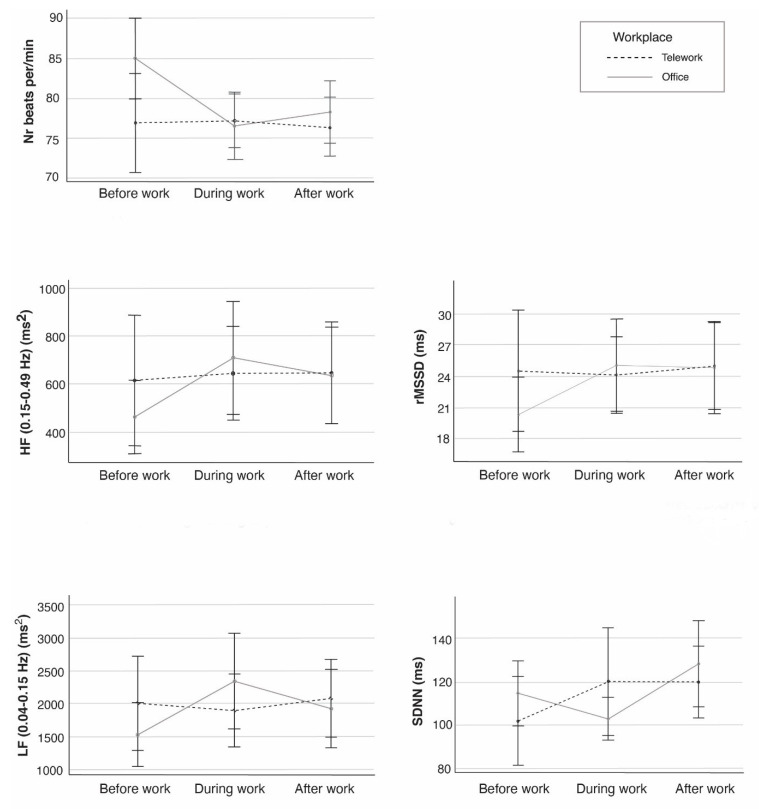
Estimated marginal means of Workplace (telework; office) and Time (leisure time before work; work during regular workhours; leisure time after work) from the repeated measure analysis of variance with heart rate (number of beats per minute) and heart rate variability indices: HF: high frequency (0.15–0.40 Hz) (ms^2^); LF: low frequency (0.04–0.15 Hz) (ms^2^); SDNN: the standard deviation of beat-to-beat intervals (ms); rMSSD (1 min intervals): the root mean squared successive differences of RRI (ms) (*n* = 20). Error bars represent 95% confidence interval.

The heart rate and heart rate variability measures generated complete data for 20 participants, data from the remaining participants were excluded due to low quality. In the repeated measures analysis of variance (*n* = 20), Workplace and Time had a significant interaction effect on heart rate (bpm), which was highest before and after workhours and slightly lower during regular workhours at the office compared to telework. For heart rate variability, a significant Workplace and Time interaction effect were seen in all variables (Table 2). Figure 2 shows the marginal means for Workplace and Time, for heart rate and each heart rate variability variable. The largest differences in psychophysiological reactivity were seen before workhours, and the smallest differences were seen after workhours (Figure 2). For teleworking days, the reactivity did not change markedly during the day, while for office days, it peaked during workhours. After adjusting the repeated measure analysis of variance for commuting time, children living at home and gender, the magnitude of the Workplace and Time interaction effect did not change. SDNN was higher and showed more variation for men (telework: M = 130.54 ms; office: M = 121.04 ms) than for women (telework: M = 97.70 ms; office: M = 108.48 ms) throughout the workday regardless of whether work was performed in the conventional office or on teleworking days. Similar results were found for rMSSD and HF.

### 3.3. Postures and Movements

The accelerometry measures generated complete data for all participants. For two participants, however, all behaviors were not present during all Workplace and Time levels and therefore they were excluded from the ilr transformations. The repeated measures analysis of variance of accelerometry measures with ilr transformed sedentary behavior relative to other behaviors showed no significant interaction effect of Workplace and Time (η_2_ = 0.013) but a significant effect of Time (η_2_ = 0.480) (Table 3). Most time (min) spent in sedentary position was seen during regular workhours and during leisure time after workhours (Table 4). In total, staff spent most time in different physical behaviors during office days, which is in agreement with previous findings [51]. There was a significant Workplace and Time interaction effect for the variation in movements, i.e., transitions between sitting and standing (η_2_ = 0.194), with more transitions being made during teleworking hours (M = 31) than during office hours (M = 25). Arm angles (50th percentile) showed no significant effect for Workplace and Time interaction (η_2_ = 0.024). For working days performed at the office and home alike, arm elevation increased over the day with lowest elevation during leisure time before workhours (telework: M = 23.93°, SD = 11.38°; office: M = 24.36°; SD = 7.18°) and most elevation after workhours (telework: M = 34.53° SD = 14.72°; office: M = 32.17°; SD = 8.87°). The magnitude of Workplace and Time interaction effects did not change after adjusting for commuting time, children living at home and gender.

## 4. Discussion

In the present study, we aimed to determine if psychophysiological reactivity, postures and movements differed during telework (i.e., work performed at home) compared to work performed at the conventional office amongst academic teaching and research staff. We found differences in all heart rate variability measures throughout the day when teleworking compared to working at the conventional office. Generally, the participants had more parasympathetic activity and higher overall heart rate variability during teleworking days compared to office days. In addition, the results showed more variation between seated and standing postures during telework than during work at the office.

### 4.1. Psychophysiological Reactivity

During telework, the participants’ heart rate variability was relatively stable over time whereas during work at the office, heart rate variability showed more variation throughout the day. The results generally displayed more parasympathetic activity (indicated by rMSSD and HF) and higher overall heart rate variability (SDNN) during teleworking days compared to workdays at the office. This may indicate that the participants are more relaxed during teleworking days than office days [37,52,53].

Previous studies have suggested that commuting struggle [10,44,45] and children living at home [14,44,46] could contribute to the level of stress among employees. In the present study, the majority of the participants had children living at home. However, neither children living at home nor commuting time affected the differences in psychophysiological reactivity found between telework and office work. It should be noted that we did not collect detailed information about whether the participants had children present at home during the studied workdays. In addition, the commuting time was relatively low in the sample.

In the present study, we found no differences between men and women in psychophysiological reactivity during telework versus office work. Rather, men had more parasympathetic activity (rMSSD, HF) and higher overall heart rate variability (SDNN) at all times at both workplaces, which may indicate more relaxation among men than among women during workdays in general. Our results stand in contrast to Lundberg and Lindfors findings [24] where elevated blood pressure after teleworking hours was found among men compared to women. The authors [24] suggested that this difference might be attributed to overtime work. In the present study, overtime work reported in the participants’ diaries averaged 4.5 h (*n* = 5) and was equal for men and women, and was excluded from leisure time after workhours. Thus, there seems to be no differences in men and women’s psychophysiological reactivity to telework versus office work when only regular workhours are considered.

Previous telework experience has been considered to provide employees with knowledge that may facilitate their teleworking practice and their health at work [5,54]. This may have affected findings in present study, as the participants were experienced teleworkers with the majority teleworking several times per month or several times per week. Furthermore, a high degree of autonomy has been shown to mediate the relationship between telework and favorable health effects by, e.g., increasing individual work control [1,5,55,56]. The participants in the present study held professions that entail high work autonomy [18,47] and thus, this may also affect the impact of telework on our sample and explain the difference between our findings and findings among other professions [24]. Psychosocial factors in the work environment (e.g., social relationships with colleagues) has previously been suggested to affect work stress and staffs’ choice to practice telework [57,58]. Since information about psychosocial factors were not collected in this study, we cannot determine whether social work relations might have affected participants’ levels of stress at the office or during telework.

For cortisol levels, we did not find any differences between telework and office work. Similarly, Lundberg and Lindfors [24] did not see any differences in cortisol levels between workplaces. This may suggest that the effects of telework on psychophysiological reactivity is not very long lasting [30,59].

Lundberg and Lindfors [24] argued that the difference in participants’ blood pressure between workplaces that they found, could be due to differences in work tasks and physical behaviors. In their study, work at the conventional office meant more frequent changes between work tasks and more engagement in physical activities, compared to teleworking. In the present study, participants’ diaries did not indicate any difference in work tasks performed during telework and office work. Their main work tasks were teaching, research and administrative activities at both workplaces. Although, we did find some differences in the measures of physical behaviors. Because it is well-established that there is an association between physical activity and psychophysiological reactivity [29,33,59,60,61], this might be a possible explanation for our findings.

### 4.2. Postures and Movements

Workplace and Time showed a significant interaction effect for the number of transitions between seated and standing postures. Participants performed more transitions during regular workhours of teleworking compared to workhours at the office. Nevertheless, the results showed that the participants generally spent much time in sedentary behaviors (i.e., sitting/standing) throughout the day at both workplaces. Physical inactivity is well documented among office workers in general [27,33,34,61], during telework [1,5] and among academic teleworkers [23]. Prolonged sedentary behaviors have been associated with musculoskeletal disorders and imbalanced autonomic regulation causing, e.g., cardiovascular disease [62,63]. Such health risks may thus be present for academic staff at either workplace.

Arm elevation did not differ between workplaces. According to previous research, the arm angles found in this study can be considered acceptable if provided with full arm support [64]. However, we do not know what the participants’ ergonomic work conditions look like. It has previously been proposed that, working from home may pose a risk for, e.g., musculoskeletal disorders due to insufficient ergonomic conditions [1,5,62,65]. Therefore, we suggest that future studies should pay more attention to teleworkers’ ergonomic prerequisites.

### 4.3. Strengths and Limitations

This study includes a homogeneous sample that could be considered representative for the population in terms of age, gender and academic title [66]. Only academic staff with previous experience of telework, who teleworked regularly was included. The disparate findings seen in previous research has partly been explained by the use of mixed samples of professions where the experience of telework has varied [1,5,9,17,31]. Because of this, it has been recommended to select professions recognized as frequent teleworkers to gain a better understanding of how this work arrangement can affect health [1,5,17,31]. The high adoption of telework among teaching and research staff, together with the insufficient knowledge of how telework affects their health, warrants studies on this population.

Lack of clear descriptions of how telework is defined has also been described as a general limitation in previous research [1,5,17,31]. Among other things, there are inconsistencies regarding from which place, and during which time, work is considered to be telework [1,5,67,68]. In the present study, telework was defined as work from home performed during regular workhours. We used diaries to identify participants’ workplace and to categorize hours of each day into regular workhours and leisure time. Additionally, we used the diaries to exclude overtime work and commuting time from those periods of regular workhours and leisure time.

To avoid potential bias in self-reports of, e.g., physical activity and stress we used objective measures to determine whether postures, movements and psychophysiological reactivity differed between days of work at the two workplaces [26,27]. We also collected information about the work tasks performed to verify the importance of work tasks in comparisons between telework and office work.

The major limitation of the present study is the small sample size, which affects the power of the statistical tests, and thus the possibility of finding significant differences. However, the within-subject analysis used in the present study may compensate to some extent for the small sample size [69,70].

Our findings represent the effects of teleworking as a voluntary choice during regular work and life conditions. Voluntariness is probably an important factor for the effects of telework as it gives staff the opportunity to adapt their choice to telework after their personal needs, which could be beneficial for their health and well-being [57,71,72]. Our findings may, therefore, stand in contrast to results from studies performed in situations where telework is forced (e.g., during the COVID-19 lockdowns) [65,73,74]. However, findings from so-called “normal” work-life conditions, with experienced teleworkers, will most likely be important for the future development of telework in working life.

## 5. Conclusions

In the present study, we found that academics had more parasympathetic activity during telework than office work, which may indicate more relaxation during teleworking hours. At the same time, they switched between sitting and standing more often during days of telework. Considering the overall large amount of time spent sedentary, this behavior may be beneficial for their health. Larger studies are needed to confirm this.

## Figures and Tables

**Table 1:** Sample characteristics (*n* = 23).

		Proportion	Mean	* SD
Age (years)			47.2	8.7
Gender	Male	48		
	Female	52		
BMI			25.4	4.8
Marital status	Living alone	4		
	Living with partner	87		
Children at home		65		
Profession	Junior lecturer	56		
	Senior lecturer	35		
	Professor	9		
Number of workdays	Office		3.2	0.5
	Telework		1.8	0.6
Frequency of telework	Less than 1 time/month	30		
	Several times/month	44		
	Several times/week	26		
Commuting time (min) (*n* = 22)			38.7	35.3

Notes. * SD: standard deviation.

**Table 2:** The Workplace (telework; office) and Time (leisure time before workhours; work during regular workhours; leisure time after workhours) effects and interaction effect for heart rate and heart rate variability measures (*n* = 20).

Variable Descriptions		F	* *p*	Partial η^2^	Power
Heart rate (bpm)	Time	3.220	0.051	0.145	0.580
	Workplace	8.557	**0.009**	0.311	0.792
	Time × Workplace	7.669	**0.002**	0.288	0.931
HF (ms^2^)	Time	3.582	**0.038**	0.159	0.629
	Workplace	1.282	0.272	0.063	0.189
	Time × Workplace	3.394	**0.044**	0.152	0.604
LF (ms^2^)	Time	4.833	**0.013**	0.203	0.767
	Workplace	1.152	0.295	1.152	0.175
	Time × Workplace	8.573	**0.001**	0.311	0.955
SDNN (ms)	Time	3.621	**0.036**	0.160	0.634
	Workplace	0.068	0.797	0.004	0.057
	Time × Workplace	3.501	**0.040**	0.156	0.618
rMSSD (ms)	Time	2.943	0.065	1.134	0.540
	Workplace	2.595	0.124	0.120	0.334
	Time × Workplace	4.412	**0.019**	0.188	0.514

Notes. bpm: beats per minute. Heart rate variability indices: HF: high frequency (0.15–0.40 Hz); LF: low frequency (0.04–0.15 Hz); SDNN: the standard deviation of beat-to-beat intervals; rMSSD (1 min intervals): the root mean squared successive differences of RRI. * Significant differences are shown in bold, *p* < 0.05.

**Table 3:** The Workplace (telework; office) and Time (leisure time before workhours; work during regular workhours; leisure time after workhours) effects and interaction effect for postures and movements (*n* = 23).

Variable Descriptions		F	* *p*	Partial η^2^	Power
Median arm angle 50 percentile	Time	10.484	**0.001**	0.333	0.969
	Workplace	1.255	0.275	0.056	0.188
	Time × Workplace	0.520	0.557	0.024	0.120
Number of transitions sit to stand	Time	63.853	**0.000**	0.753	1.000
	Workplace	0.036	0.851	0.002	0.054
	Time × Workplace	5.059	**0.021**	0.194	0.688
CODA, Ilr (*n* = 21)	Time	18.432	**0.000**	0.480	1.000
	Workplace	0.001	0.972	0.000	0.050
	Time × Workplace	0.262	0.771	0.013	0.088

Notes. Ilr: the isometric log-ratio coordinate of the relative information between sedentary behaviors and other behaviors (i.e., standing/walking/moving). * Significant differences are shown in bold, *p* < 0.05.

**Table 4:** Estimated geometrical means for time (min) spent in different physical behaviors before, during and after workhours on teleworking days and office days (*n* = 23).

Variable Descriptions		Before Work	During Work	After Work	Total Time
Sit/lie	Telework	34	324	193	551
	Office	41	312	222	575
Stand	Telework	20	78	57	155
	Office	20	73	64	157
* Move	Telework	15	66	54	135
	Office	26	51	66	143

Notes. * Physical movements such as walking, running, cycling.

## Data Availability

The datasets generated and analyzed during the current study are not publicly available but are available from the corresponding author on reasonable requests.
